# Seemingly altruistic behavior and strategic ignorance in a dictator game with potential loss

**DOI:** 10.3389/fpsyg.2024.1473500

**Published:** 2025-01-10

**Authors:** Keisuke Yamamoto, Hirofumi Hashimoto

**Affiliations:** ^1^Department of Social Relations, Faculty of Social Relations, Kyoto Bunkyo University, Uji, Japan; ^2^Graduate School of Literature and Human Sciences, Osaka Metropolitan University, Osaka, Japan

**Keywords:** dictator game, strategic ignorance, empathy-altruism hypothesis, empathic concern, potential loss

## Abstract

Previous studies have indicated that in the standard binary version of the dictator game, people are less likely to behave altruistically when given the opportunity to be strategically ignorant. The present study aims to assess the robustness of individuals’ strategic ignorance in the context of the emergence of empathic concern. It is reasonable to hypothesize that traditional standard dictator games may not be an optimal context for the emergence of empathic concern. Accordingly, the purpose of the present study is to use a dictator game with loss potential that facilitates player empathic concern to investigate the emergence of (seemingly) altruistic behavior and strategic ignorance in the context of empathy. The results of a web experiment with 200 Japanese adults showed that our manipulation of loss had no effect on the occurrence of altruistic behavior and strategic ignorance. Moreover, even in situations in which the participant, as a dictator, is aware that the recipient has suffered a loss, they behave as if they have a legitimate reason not to act altruistically by being strategically ignorant. This result demonstrates the robustness of strategic ignorance and indicates that evoking empathic concern may not have enough of an effect to influence altruistic behavior in the laboratory experiment.

## 1 Introduction

Empirical studies in social psychology and behavioral economics indicate that human beings frequently engage in decision-making that prioritizes altruistic considerations over economic rationality. For instance, in dictator games, which experimentally examine people’s altruism, an allocator is free to distribute a certain amount of money, while the recipients passively accepts the allocator’s decision without any right to influence the payoff distribution. Findings from such games reveal that allocators give approximately 28% of the original sum on average, with one-sixth of allocators distributing half of their money (Camerer, 2003). However, do this behavior reflect genuine altruism, according to which individuals care for others?

Batson (2010) proposed the empathy–altruism hypothesis, which suggests that altruistic behaviors are motivated by empathic concern—emotional reactions of sympathy and care for others—when individuals perceive others’ state of neediness. In standard dictator games, the recipients are typically not portrayed as being in need, with the notable exception of the study by Brañas-Garza (2007). When recipients are not perceived as being in need, empathic concern is less likely to arise, potentially leading to an underestimation of altruism in standard dictator games. Therefore, this study seeks to determine whether altruistic behavior in a dictator games increases when recipients are in a state of need.

Similarly, the role of reputation management warrants discussion in light of findings from social psychology. Previous research using dictator games has examined how contexts on reputation influence altruistic and selfish behaviors. Hoffman et al. (1994) identified an anonymity effect, whereby the amount allocated to recipients decreases as the allocator’s anonymity increase. Findings by Dana et al. (2006) and Lazear et al. (2012) indicate that allocators give less when they can exit the game and keep their payoffs in secrecy without the recipient’s knowledge. Moreover, Dana et al. (2007) found that giving is lower when the allocators can voluntarily reveal the recipient’s payoff, because they are unaware of the payoff outcome, even if they act selfishly.

Examining these contextual effects on reputation provides valuable insights into the impact of empathy, which is the key area of interest in this study. The present study aims to explore altruism in dictator games by investigating whether the contextual effects on reputation are robust not only in standard dictator games, but also when recipients are in need. To this end, the study focuses on empathy, and reputation management.

### 1.1 Strategic ignorance in the dictator game

As noted above, while several contextual effects drive individuals to behave selfishly, the present study focused specifically on strategic ignorance as observed in Dana et al.’s (2007) experiment. This serves as an initial exploration of the robustness of reputation effects in different contexts. Dana et al.’s (2007) study employed a binary version of the dictator game, wherein participants (allocators) were required choose between two payoff options. Two conditions were included: one, where both the allocator’s and recipient’s payoffs were disclosed (baseline), and another where the allocators could voluntarily reveal the recipient’s payoff (hidden information treatment). For instance, in the baseline, participants were explicitly informed that choosing a$5 payoff for themselves would result in the recipient also receiving $5, while choosing a $6 payoff for themselves would leave the recipient with only $1. In the hidden information treatment, allocators initially knew only their own payoff, and could choose to reveal the recipient’s payoff. Results revealed that 63% of the percentage of participants in hidden information treatment opted for the selfish choice (earning more for themselves), compared to 26% in the baseline. Furthermore, 56% of participants in the hidden information treatment chose to reveal the recipient’s payoff. One plausible explanation for this behavior is that participants deliberately avoided learning the recipient’s payoff to justify their actions, claiming ignorance of the recipient’s situation. This behavior is known as *strategic ignorance*. Subsequent studies have successfully replicated Dana et al.’s findings (Grossman, 2014; Larson and Capra, 2009), demonstrating the reliability of this phenomenon.

Moreover, research (Grossman, 2014) has demonstrated that the prevalence of strategic ignorance can be influenced by altering the experimental context or introducing additional manipulations. Grossman (2014) replicated Dana et al.’s (2007) results and demonstrated that changing specific experimental setting reduced the occurrence of strategic ignorance. In Dana et al.’s (2007) original setup, default condition (default NR condition) withheld the recipient’s payoff unless participants pressed a “reveal game” button. Grossman (2014) introduced an active choice condition that explicitly asked participants whether they wanted to reveal the recipient’s payoff (“yes” or “no”). This adjustment heightened the salience of the participant’s intentional disregard for the recipient’s payoff. As a result, the prevalence of strategic ignorance decreased from 42% in the default NR condition to 25% in the active choice condition.

Nevertheless, no study has yet investigated the occurrence of strategic ignorance in contexts where empathic concerns are elicited. Strategic ignorance (or the decision to reveal a recipient’s payoff) can also serve as an indicator of attention to others, offering insights that are qualitatively distinct from allocation behaviors in dictator games. To further support the validity of the empathy-altruism hypothesis, this study examines not only on the allocation decision, but also the phenomenon of strategic ignorance. According to Batson’s (2010) empathy-altruism hypothesis, individuals experiencing empathic concern are motivated to act with the goal of improving the recipient’s state of need. Achieving this goal requires analyzing the extent of the recipient’s impoverishment and determining the level of assistance required. Therefore, in contexts where empathic concern is activated, it is hypothesized that individuals will be less inclined to employ strategic ignorance and more likely to reveal the recipient’s payoff to accurately assess their situation.

### 1.2 Effects of potential loss

The majority of studies utilizing dictator games have not incorporated manipulations of the recipient’s needs, with two notable exceptions: Goto (2018) and Brañas-Garza (2007), both of which addressed the recipients’ needy situations. In Goto’s (2018) experiment, the participants first completed five rounds of a public goods game. After these rounds, two out of four players were randomly selected, and their earnings were multiplied by 0.3. Players whose earnings were reduced in this manner were assumed to have suffered a loss and to be more impoverished. Conversely, players whose earnings were not reduced were considered less impoverished. Following this, players participated in a dictator game involving three other players. Results demonstrated that allocators gave more money to recipients who had experienced losses. Although not directly involving dictator games, Barclay and Benard (2020) also examined the effects of potential loss using public goods games. In their experiment, participants played a public goods game under the threat that all players’ earnings could be reduced to zero with a random probability. This probability could be mitigated by players making decisions that contributed to the group. The results indicated that the higher the perceived probability of a threat, the more likely participants were to contribute to the group.

Brañas-Garza (2007) did not manipulate player’s rewards but instead used framing to highlight the recipients’ needy status in the dictator game. In the baseline, participants were provided with instructions explaining the number of trials, the roles of allocator and recipient, and the pair matching procedure. In the framing condition, the same instructions were presented, with the addition of final sentence: “Note that your recipient relies on you.” This framing manipulation aimed to draw participants’ attention to a specific moral obligation. As a result, donations in the framing condition were higher than in the baseline.

The studies by Barclay and Benard (2020), Goto (2018), and Brañas-Garza (2007) did not specifically examine whether loss manipulation elicited empathic concern. However, their findings can be interpreted within the framework of the empathy-altruism hypothesis. That is, when the recipient’s payoff was reduced or at risk of being reduced, participants exhibited empathic concern and were more likely to engage in altruistic behavior. Therefore, the present study employed these potential loss manipulations to evoke recipients’ needs and eliciting empathic concern.

### 1.3 Empathy-altruism hypothesis

As mentioned earlier, the empathy-altruism hypothesis proposed by Batson (2010) posits that altruistic behavior is motivated by a genuine concern for others. However, Archer et al. (1981) offers a different perspective on the relationship between empathy and altruistic behavior. The empathy-specific evaluation hypothesis suggested by Archer et al. (1981) argues that people may perform altruistic behavior not out of pure empathy but to avoid negative judgment from others. In this view, individuals act altruistically to manage their reputation, as they have learned that failing to do so when empathy is elicited can lead to blame. Thus, according to the empathy-specific evaluation hypothesis, the relationship between empathic concern and altruistic behavior is ultimately driven by a selfish desire to protect one’s reputation.

To test the empathy-altruism hypothesis, Fultz et al. (1986) conducted an experiment where the amount of time spent with a lonely recipient (a girl) was used as an indicator of altruistic behavior. In the high-empathy condition, participants were instructed to imagine how the recipient felt. In the low-empathy condition, participants were instructed to adopt an objective perspective. In the high-social evaluation condition, participants’ behaviors were visible to others, whereas in the low-social evaluation condition, their behaviors were not observed by others. If the empathy-altruism hypothesis holds, it predicts that high empathy would lead to the same degree of altruistic behavior, regardless of social evaluation. The results demonstrated that only the main effect of empathy was significant, indicating that higher empathy led to more helping behavior. Fultz et al. (1986) concluded that this supports the empathy-altruism hypothesis.

However, the experiment by Fultz et al. (1986) has several limitations. The perspective-taking manipulation to evoke empathic concern may not have been robust. McCauley et al. (2024) conducted a follow-up study, but the perspective-taking manipulation did not work as intended, failing to replicate the results. This suggests that guiding participants’ perspective-taking through the experimenter’s instructions may be challenging.

Given these limitations, we decided to test the empathy-altruism hypothesis using methods alternative to those employed by Fultz et al. (1986). Instead of perspective-taking, this study used the manipulation of potential loss to evoke empathic concern. While perspective-taking relies on the subjective effort of the participant, the manipulation of potential loss is more objective, as it creates a situation in which the recipient is in need. This approach may evoke stronger empathic concern. Additionally, although this study did not included social evaluation as an experimental manipulation, the occurrence of strategic ignorance in the dictator game can serve as an indicator of reputation management. Thus, this study aims to test the empathy-altruism hypothesis by comparing the occurrence of strategic ignorance in the standard dictator game with that in the dictator game where potential loss is present.

In summary, this study aims to evaluate the robustness of strategic ignorance in a dictator game incorporating potential loss. Based on the empathy-altruism hypothesis (Batson, 2010), we hypothesize that, in a dictator game involving losses, allocators will be more likely to reveal the recipient’s payoffs and give more money compared to the standard dictator game, due to the emergence of empathic concern.

## 2 Method

### 2.1 Participants

Two hundred Japanese participants (100 males and 100 females, *M_age_* = 45.32 ± 13.49) who had registered through an online panel maintained by a marketing research firm (MyVoice) were recruited for this study and participated in the online experiment conducted in early October 2023. All participants were included in the analysis and experimental payoffs were awarded based on their decisions.

### 2.2 Experimental design

This experiment utilized a two-factor, between-participants design. The first factor involved the manipulation of potential loss. In the loss condition, the recipient’s payoff was reduced, while in the control condition, it was not. The second factor involved the manipulation of hidden information. In the baseline condition, the recipient’s payoffs were disclosed to the allocator from the beginning. In the hidden information treatment, the recipients’ payoffs were initially concealed, and the allocator could choose to reveal them before making a decision.

### 2.3 Procedure

After receiving an overview of the experiment, participants completed a memory task. To manipulate potential loss, participants earned payoffs prior to the dictator game. They were instructed to remember the order of 10 different figures that were displayed on a screen for 3 min, after which they were informed that they would receive a payoff of ¥500 for completing the task.

Following the memory task, participants were told that they would be paired with another participant for the next task. They were also informed that they would not know the identity of the paired partner, as the pairing would be determined randomly.

### 2.4 Manipulation of loss

Participants were informed that they and their paired partner would each draw lots, and the results would determine whether their ¥500 payoff from the memory task would be reduced. In the lottery, participants selected one of the eight cards that had been turned over. If a participant drew a white card with ¥500 written on it, they could retain their full ¥500 payoff. However, if they drew the card was a red card with ¥100 written on it, their ¥500 payoff would be reduced to ¥100 (a loss of ¥400). After the draw, participants were provided feedback on their own payoffs as well as those of their paired partner. In practice, all participants drew white cards, retaining the full ¥500. The loss manipulation was implemented by informing the participants whether their paired partner (the recipient) retained their full ¥500 (control condition) or had their payoff reduced to ¥100 (loss condition).

### 2.5 The dictator game

Participants were briefed on the dictator game. They were informed that, by completing the decision-making task (the dictator game), they would earn a new payoff, separate from the payoff from the memory task. In the decision-making task, one role was the allocator, who was responsible for selecting the payoff; the other role was the recipient, who received the payoff determined by the allocator. Participants were told that their role would be assigned based on their selection of one of the two cards presented on the screen.^1^ However, in practice, all participants were assigned to the allocator role. Once informed of their role, participants chose one of three payoff options: “a. You: ¥400/partner: ¥100,” “b. You: ¥250 / partner: ¥250,” or “c. You: ¥100/partner: ¥400.”

### 2.6 Manipulation of hidden information

**Figure 1:**
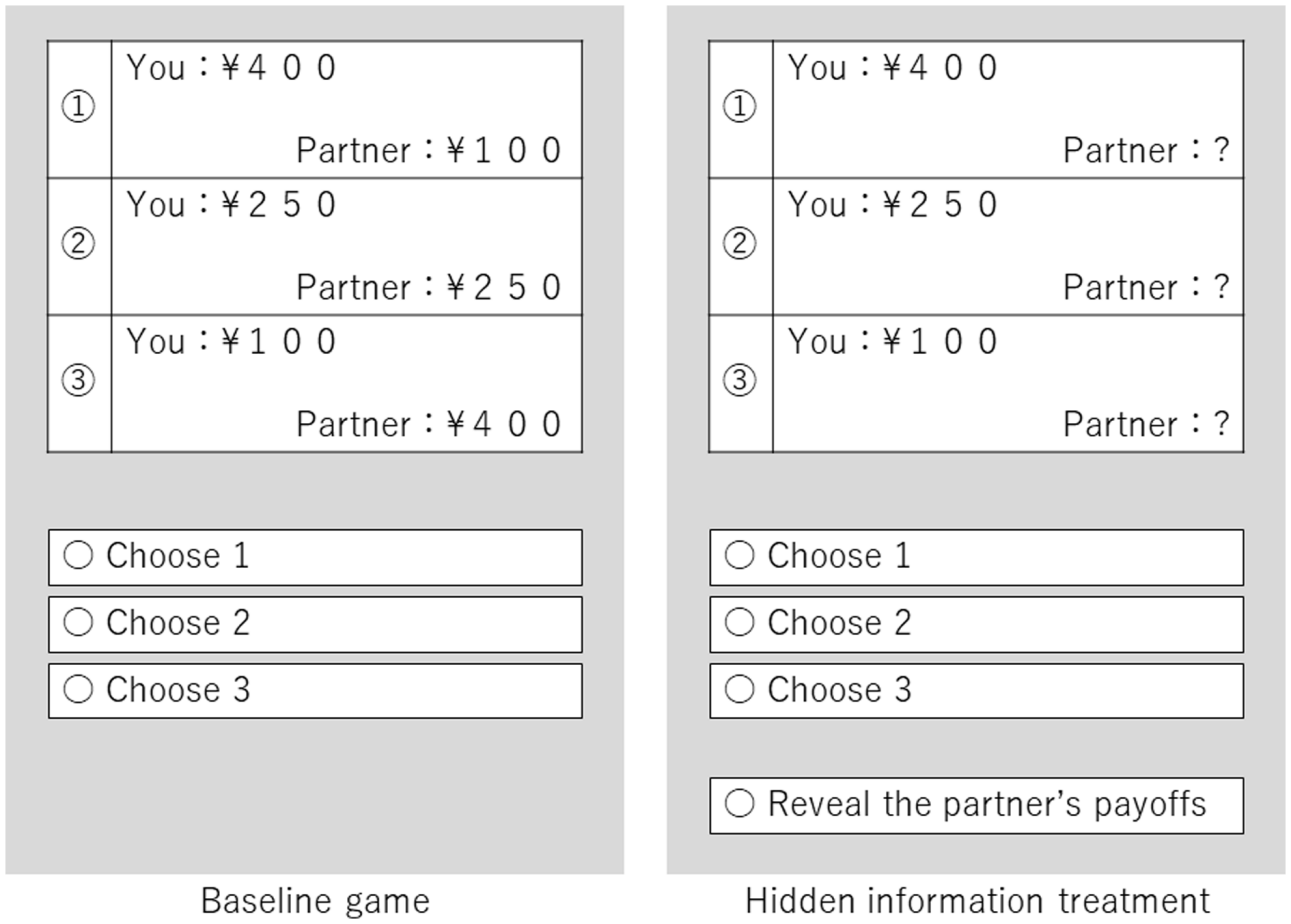
Decision screen in each treatment.

In the baseline, the payoffs earned by the paired partner (the recipient) were disclosed to the participants from the beginning. In the hidden information treatment, participants were initially unaware of the recipient’s payoff. If participants chose to “reveal the partner’s payoffs,” the recipient’s payoff was disclosed; otherwise they could complete the task without revealing the recipient’s payoff. Figure 1 illustrates the decision screen for each condition.

### 2.7 Questionnaire

Participants were asked to respond to a questionnaire about their thoughts during the decision-making task. The first scale assessed empathic concern, and the second measured evaluation concerns. The empathic concern scale was adapted from items used by Fultz et al. (1986) for manipulation checks. It was measured with four items: “I felt sympathy for the paired partner,” “I felt compassion for the paired partner,” “I felt empathy for the paired partner,” and “I felt concern for the paired partner.” The second scale, measuring evaluation concerns, included three items: “I was concerned that my behavior would be known by others,” “I was trying to avoid negative evaluation from others,” and “I was worried that my reputation would be damaged.” This scale was developed Based on Archer et al.'s (1981) assumption of a selfish desire to avoid a bad reputation.

Finally, participants were asked to provide their sex and age. They were then debriefed, informed that there were other experimental conditions, and that the paired partners were fictitious.

## 3 Results

Descriptive statistics are indicated in Table 1. Participants had three choices in the decision-making task: “a. You: ¥400/partner: ¥100 (selfish choice),” “b. You: ¥250/partner: ¥250 (equitable),” or “c. You: ¥100/partner: ¥400 (altruistic choice).” Since very few participants chose the altruistic choice across the conditions, the analysis for hypothesis testing combined the equal and altruistic choices into a single category, referred to as the variable “binary payoff distribution” (selfish vs. equal and altruistic choices).

**Table 1:** Descriptive statistics.

	Control			Loss		
	Baseline	Hidden information		Baseline	Hidden information	
		No revealing	Revealing		No revealing	Revealing
*N*	50	30	20	50	31	19
Payoff distribution						
Selfish	13 (26.00%)	14 (46.70%)	6 (30.00%)	17 (34.00%)	19 (61.30%)	6 (31.60%)
Equal	34 (68.00%)	13 (43.30%)	11 (55.00%)	29 (58.00%)	12 (38.70%)	12 (63.20%)
Altruistic	3 (6.00%)	3 (10.00%)	3 (15.00%)	4 (8.00%)	0 (0.00%)	1 (5.30%)
Binary payoff distribution						
Selfish	13 (26.00%)	14 (46.67%)	6 (30.00%)	17 (34.00%)	19 (61.29%)	14 (31.58%)
Equal and altruistic	37 (74.00%)	16 (53.33%)	14 (70.00%)	33 (66.00%)	12 (38.71%)	13 (68.42%)
Empathic concern						
*M*	3.56	3.48	3.55	3.62	3.21	3.34
*SD*	1.31	1.06	1.74	1.33	1.40	1.34
Evaluation concern						
*M*	3.45	3.23	3.15	3.35	3.22	2.82
*SD*	1.52	1.23	1.46	1.44	1.34	1.40

### 3.1 Manipulation check

A two-factor analysis of variance (ANOVA) was conducted to confirm whether the manipulation of loss evoked empathic concern. The results demonstrated no significant main effect of loss [*F*(1, 196) = 0.24, *p* = 0.63, *_partial_ η*^2^ = 0.00], no significant main effect of hidden information [*F*(1, 196) = 1.14, *p* = 0.29, *_partial_ η*^2^ = 0.01], and no significant interaction [*F*(1, 196) = 0.65, *p* = 0.42, *_partial_ η*^2^ = 0.00]. Thus, we could not confirm whether the manipulation of loss successfully evoked empathic concern.

A two-factor ANOVA was conducted to assess whether the manipulation of hidden information caused differences in evaluation concerns. The results indicated that neither the main effect of loss [*F*(1, 196) = 2.78, *p* = 0.10, *_partial_ η*^2^ = 0.01], the main effect of hidden information [*F*(1, 196) = 0.37, *p* = 0.54, *_partial_ η*^2^ = 0.00], nor the interaction [*F*(1, 196) = 1.41, *p* = 0.24, *_partial_ η*^2^ = 0.01] were significant.

However, for participants who revealed the recipients’ payoffs in the hidden information treatment, the condition was the same as at the baseline. Therefore, instead of using the hidden information experimental condition as the independent variable, a two-factor ANOVA was conducted using data from the hidden information treatment only, with the independent variable being whether the participant revealed the payoff.

When empathic concern was the dependent variable, the analysis revealed no significant main effect of loss [*F*(1, 96) = 0.70, *p* = 0.40, *_partial_ η*^2^ = 0.00], no significant main effect of revealing payoffs [*F*(1, 96) = 0.14, *p* = 0.71, *_partial_ η*^2^ = 0.00], no significant interaction [*F*(1, 96) = 0.01, *p* = 0.92, *_partial_ η*^2^ = 0.00]. Similarly, when evaluation concern was the dependent variable, there were no significant main effects of loss [*F*(1, 96) = 0.39, *p* = 0.53, *_partial_ η*^2^ = 0.00], revealing payoffs [*F*(1, 96) = 0.74, *p* = 0.39, *_partial_ η*^2^ = 0.01], or the interaction [*F*(1, 96) = 0.31, *p* = 0.58, *_partial_ η*^2^ = 0.00]. Therefore, the psychological scale data did not confirm whether the experimental manipulations were effective.

### 3.2 Hypothesis testing

A chi-square test was conducted to examine whether the proportion of participants revealing payoffs differed between the control and loss conditions. Table 2 presents the frequencies and percentages for each condition. The results of the chi-square test were not significant [*χ*(1) = 0.04, *p* = 0.84], indicating that the percentage of participants revealing payoffs did not differ between the control and loss conditions.

**Table 2:** Cross table on revealing payoffs and loss potential.

	Control	Loss
Revealing payoffs		
No revealing	30 (60.00%)	31 (62.00%)
Revealing	20 (40.00%)	19 (38.00%)

Only the hidden information treatment (*N* = 100) were used in the analysis.

A logistic regression analysis was conducted to explore the effects of loss manipulation and hidden information manipulation on the binary payoff distribution (selfish vs. equal and altruistic choices). The results are summarized in Table 3. The sex of the participants was also included as a covariate in the model. The main effect of hidden information was significant (odds ratio = 0.52, 95% CI [0.29, 0.93], *p* = 0.03), indicating that equal and altruistic choices were more likely to be made under the baseline than under the hidden information treatment. Neither the main effect of loss (odds ratio = 0.67, 95% CI [0.37, 1.21], *p* = 0.18), the interaction between hidden information and loss (odds ratio = 0.98, 95% CI [0.30, 3.18], *p* = 0.97), nor the main effect of sex (odds ratio = 0.56, 95% CI [0.31, 1.01], *p* = 0.06) had a significant effect on the binary payoff distribution.

**Table 3:** Logistic regression analysis of the effects of loss and hidden information manipulations.

Dependent variable: Binary payoff distribution	Odds ratio	95% confidence interval	
		Lower limit	Upper limit
Sex	0.56	0.31	1.01
Loss	0.67	0.37	1.21
Hidden information	0.52*	0.29	0.93
Loss × Hidden information	0.98	0.30	3.18

^*^p < 0.05.

In the hidden information treatment, participants who revealed their paired partners’ payoffs were essentially the same as those in the baseline. Therefore, only the hidden information treatment (*N* = 100) was included in the analysis, with revealed payoffs, losses, and sex as independent variables. The results are summarized in Table 4. The main effect of revealing the payoff was significant (odds ratio = 2.43, 95% confidence interval [CI] [1.02, 5.79], *p* < 0.05), suggesting that participants who revealed their partner’s payoff were more likely to make equal and altruistic choices compared to those who did not reveal the payoff. The main effect of sex was also significant: female participants were more likely to make equal and altruistic choices than male participants (odds ratio = 0.43, 95% confidence interval [CI] [0.19, 1.00], *p* < 0.05). Neither the main effect of loss (odds ratio = 0.71, 95% confidence interval [CI] [0.30, 1.68], *p* = 0.43) nor the interaction between revealing payoffs and loss was significant (odds ratio = 1.78, 95% confidence interval [CI] [0.31, 10.08], *p* = 0.52).

**Table 4:** Logistic regression analysis of the effects of loss and revealing payoffs.

Dependent variable: Binary payoff distribution	Odds ratio	95% confidence interval	
		Lower limit	Upper limit
Sex	0.43*	0.19	1.00
Loss	0.71	0.30	1.68
Revealing payoffs	2.43*	1.02	5.79
Loss × Revealing payoffs	1.78	0.31	10.08

^*^p < 0.05.

## 4 Discussion

The present study revealed no significant difference between the control and loss conditions in the percentages of participants who revealed payoffs or chose equal and altruistic options. Furthermore, the results of the manipulation check indicate that the manipulation of loss did not evoke empathic concern. As a result, this study was unable to test the empathy-altruism hypothesis. Specifically, the lack of significant differences between the control and loss conditions suggests that the manipulation of loss failed to evoke empathic concern, and, therefore, the hypotheses based on the empathy-altruism framework were not supported. However, the present study did demonstrate that strategic ignorance occurs robustly, even in contexts where the recipient is in need due to financial loss. Although several research has explored the robustness of strategic ignorance (e.g., Grossman, 2014; Larson and Capra, 2009), it has not been clear whether strategic ignorance arises when the recipient is in need. This study provides new findings on the persistence of strategic ignorance, even in these needy contexts.

The present study’s finding that the manipulation of loss has no significant effect on altruistic behavior appears inconsistent with prior studies (Barclay and Benard, 2020; Brañas-Garza, 2007; Goto, 2018). For instance, Goto (2018) and Barclay and Benard (2020) demonstrated that people are more likely to behave altruistically or cooperatively when they become aware that others are experiencing a loss or are likely to lose payoffs. However, there are key differences between these previous studies and the present one.

First, Barclay and Benard (2020) investigated public goods games, which measure cooperative rather than purely altruistic behavior. In such games, contributing to a group when facing a potential loss aligns with an individual’s self-interest.

Second, Goto’s (2018) experiment involved participants experiencing reductions in their own gains while playing the public goods game before moving on to the dictator game. This shared experience may have fostered a sense of in-group membership, as it has been reported that people tend to show greater empathy toward in-group members (Avenanti et al., 2010). In contrast, participants in the present study performed the memory task individually and experienced reductions in payoffs without engaging in cooperative interactions, which may have limited the emergence of empathic bonds.

Third, both Goto (2018) and Barclay and Benard (2020) implemented actual reductions in participants’ rewards as part of the loss manipulation. In contrast, this study used deception, presenting fictitious scenarios of loss. Although it is unclear whether participants suspected this deception, any doubts could have influenced their behavior and affected the results.

Finally, Brañas-Garza (2007) employed framing manipulation to draw participants’ attention to moral rules, rather than focusing on a situation where recipients lost money. Unlike scenarios where recipients lose money due to misfortune—where the responsibility to help may seem less immediate—framing manipulations highlight the obligation to donate, potentially encouraging altruistic behavior.

These methodological differences likely contribute to the observed inconsistencies between the findings of the present study and those of earlier research.

Although the empathy-altruism hypothesis posits that perceiving others in need evokes empathic concern, which in turn motivates altruistic behavior, the present study suggests that the relationship between perceiving need and empathic concern may not be as robust as Batson (2010) claims. This conclusion, however, assumes that participants in this study did not suspect the deception inherent in the loss manipulation. Previous research has demonstrated that empathy is more likely to arise for close others (Beckes et al., 2013), highly similar others (Batson et al., 1981), and in-group members (Avenanti et al., 2010). To effectively evoke empathic concern when others are perceived as being in need, these factors may need to be carefully considered.

Nevertheless, several experiments testing the empathy-altruism hypothesis have not explicitly accounted for the relationship between the actor and the recipient. Yet, these studies have often yielded findings consistent with the hypothesis (Batson, 2010). Notably, prior research frequently employed more realistic helping scenarios rather than dictator games. For instance, studies have involved helping tasks such as spending time with a lonely girl taking on the role of another person receiving electric shocks. These contexts inherently emphasize the misfortune or dire circumstances of the individuals in need, potentially making them more effective at eliciting empathic concern. To replicate such outcomes within the framework of a dictator game, future research may need to design scenarios that more strongly emphasize the recipient’s misfortune or precarious situation.

Moreover, it is important to highlight the intersection between the morality preference hypothesis and the empathy-altruism hypothesis, as discussed earlier in this study. The morality preference hypothesis posits that decisions in dictator games are guided by a preference for doing what is morally right, rather than minimizing inequity or maximizing efficiency (Biziou-Van-Pol et al., 2015; Capraro and Perc, 2021; Capraro and Rand, 2018; Tappin and Capraro, 2018). In the absence of explicit instructions, moral judgments are influenced by individuals’ personal values. However, prior research on the morality preference hypothesis has not explicitly examined the potential link between moral judgments and empathic concern.

It seems reasonable to posit that when empathic concern is present, individuals may be more inclined to perceive helping the recipient as the morally appropriate course of action. Based on the findings of the morality preference hypothesis, it can be predicted that altruistic behavior will increase when a recipient’s needy state is explicitly established in the dictator game. If the results of this study suggest that participants’ perception of giving as a morally appropriate action does not differ when the recipient is in need, this may offer intriguing insights into the morality preference hypothesis. Although it is evident that the experimental design of this study does not allow for definitive conclusions, further research is warranted to explore the effects of loss on distribution amounts and the occurrence of strategic ignorance in the dictator game.

Several unresolved issues merit attention. Firstly, the study demonstrates inconsistencies regarding sex differences. Specifically, analysis of the hidden information treatment (N = 100) showed that females were significantly more altruistic than males. However, in the overall analysis (N = 200), which included the baseline, the effect of sex was only marginally insignificant. The disappearance of the effect in the overall analysis is unclear, though the significant finding within the hidden information treatment aligns with prior research (Engel, 2011; Rand et al., 2016; Brañas-Garza et al., 2018).

Secondly, the study’s most significant limitation lies in its inability to provide evidence that the loss manipulation successfully evoked empathic concern. This may stem from an insufficiently robust manipulation or participant skepticism regarding the deception, which undermined the conditions necessary for empathy to operate effectively. Nevertheless, the findings indicate that individuals proactively devise justifications to avoid altruistic actions, even when faced with clear evidence of others’ needs. Thirdly, the sample size in this study was determined by budgetary constraints, rather than a power analysis. As a result, the appropriateness of the sample size should be interpreted with caution.

Despite these limitations, this study attempts to contribute to the literature by examining whether “loss” influences the incidence of strategic ignorance. While the dictator game has traditionally been used to study altruistic behavior, future research should more closely investigate the role of empathy in this context.

## Data Availability

The raw data supporting the conclusions of this article will be made available by the authors, without undue reservation.

## Footnotes

^1^The explanation of card selection for determining roles in the dictator game was presented to participants independently, on a separate page from the card selection used to manipulate losses.
